# Impact of the COVID-19 Pandemic on the Intensity of Health Services Use in General Practice: A Retrospective Cohort Study

**DOI:** 10.3389/ijph.2021.635508

**Published:** 2021-05-07

**Authors:** Yael Rachamin, Oliver Senn, Sven Streit, Julie Dubois, Michael J. Deml, Katharina Tabea Jungo

**Affiliations:** ^1^ Institute of Primary Care, University of Zurich and University Hospital Zurich, Zurich, Switzerland; ^2^ Institute of Primary Health Care (BIHAM), University of Bern, Bern, Switzerland; ^3^ Department of Community Health, Institute of Family Medicine, University of Fribourg, Fribourg, Switzerland; ^4^ Department of Sociology, Institute of Sociological Research, University of Geneva, Geneva, Switzerland; ^5^ Graduate School for Health Sciences, University of Bern, Bern, Switzerland

**Keywords:** COVID-19, SARS-CoV-2, health services use, general practice, primary care, chronic conditions, risk groups, Switzerland

## Abstract

**Objectives:** We aimed to explore the impact of the Swiss shutdown in spring 2020 on the intensity of health services use in general practice.

**Methods:** Based on an electronic medical records database, we built one patient cohort each for January-June 2019 (control, 173,523 patients) and 2020 (179,086 patients). We used linear regression to model weekly consultation counts and blood pressure (BP) and glycated hemoglobin (HbA1c) measurement counts per 100 patients and predicted non-shutdown values. Analyses were repeated for selected at-risk groups and different age groups.

**Results:** During the shutdown, weekly consultation counts were lower than predicted by −17.2% (total population), −16.5% (patients with hypertension), −17.5% (diabetes), −17.6% (cardiovascular disease), −15.7% (patients aged <60 years), −20.4% (60–80 years), and −14.5% (>80 years). Weekly BP counts were reduced by −35.3% (total population) and −35.0% (hypertension), and HbA1c counts by −33.2% (total population) and −29.8% (diabetes). *p*-values <0.001 for all reported estimates.

**Conclusion:** Our results document consequential decreases in consultation counts and chronic disease monitoring during the shutdown. It is crucial that health systems remain able to meet non-COVID-19-related health care needs.

## Introduction

When the first wave of the current coronavirus disease 19 (COVID-19) pandemic swept throughout the world in spring 2020, many countries introduced mitigation measures aiming to combat the spread of the novel virus (SARS-CoV-2). In Switzerland, this initially entailed temporary prohibition of public gatherings and the closing of schools, restaurants, bars, entertainment and leisure facilities, and all shops except grocery stores and pharmacies^1^.

Whereas the public health focus has justifiably attended to immediate threats posed by SARS-CoV-2, the pandemic is likely to also have had an impact on patients and their health services use with regard to non-COVID-19 conditions and regular care, especially in general practice. From 16 March 2020 to 26 April 2020, all non-urgent treatments and consultations were banned in order to free up consultation hours in the expectation of a high workload from COVID-19 positive patients^2^. However, when this ban was in place, health care professionals expressed concerns that patients would also avoid contacting the health care system for what would be considered urgent. For example, data on hospital admissions showed a hiatus of patients with heart problems [1]. Reasons for this were presumably multifaceted. Patients might not have received clear communication about what would be considered “urgent”, which likely resulted in patients misperceiving general practice health services as being restricted to COVID-19-related concerns [2]. Furthermore, patients might have been afraid of getting infected with COVID-19 at the doctor’s office, even after the ban was lifted.

People designated to be at high risk for severe COVID-19, including adults with high blood pressure, diabetes, and cardiovascular disease^3^, require continuous treatment and regular monitoring in general practice. This renders them particularly vulnerable to health care challenges and mortality when the health care they rely on is no longer readily accessible [3, 4]. For example, even during the ban on general practice health services, patients treated with anticoagulants or requiring diabetes monitoring were considered urgent cases and encouraged to visit their GP^4^.

Thus, understanding the impact of COVID-19 on regular health care in general practice and in particular for at-risk patients is essential, especially as clinicians and the health care system continue to be confronted by subsequent waves of outbreak. However, the impact of COVID-19 on regular care is difficult to quantify. One approach is to measure the intensity of health care use by considering consultation frequencies. Accordingly, several studies from different countries have shown a decrease in consultations to emergency departments and specialists as well as a decline in the use of preventive and elective care during spring 2020 [5–12]. However, this phenomenon has been understudied in general practice, especially for at-risk groups. For these groups, frequencies of disease-specific measurements commonly used in monitoring, such as glycated hemoglobin (HbA1c) for diabetes or blood pressure (BP) for hypertension, are likely good indicators of patients receiving regular care.

In this study, we aimed to explore the impact of the COVID-19 pandemic on the intensity of health care use in general practice by investigating trends in weekly consultation- and chronic disease measurement counts for the total general practice population and for different at-risk patient groups (hypertension, diabetes, and cardiovascular disease) and different age groups.

## Methods

### Design, Setting, and Participants

This was a retrospective cohort study based on anonymized electronic medical records from Swiss general practice between January and June (calendar weeks 2–26) of the years 2019 and 2020. Data was provided by general practitioners (GP) participating in the FIRE project. FIRE stands for “Family medicine International Classification of Primary Care (ICPC) Research using electronic medical records (EMRs)”. The project currently collects routine data from EMRs from over 500 GPs, primarily in the German-speaking part of Switzerland. The FIRE project has been described in detail elsewhere [13]. In short, it contains data on consultations, medication prescriptions, laboratory and vital sign measurements, and diagnosis codes according to the ICPC 2^nd^ edition (ICPC-2), linked to the patient, GP and practice. The local Ethics Committee of the Canton of Zurich approved studies within the FIRE project (BASEC-Nr. Req-2017–00797) and waived the requirement to obtain patients’ informed consent since the FIRE project is outside the scope of the Human Research Act. We reported the study findings in accordance with the STROBE checklist [14].

**FIGURE 1:**
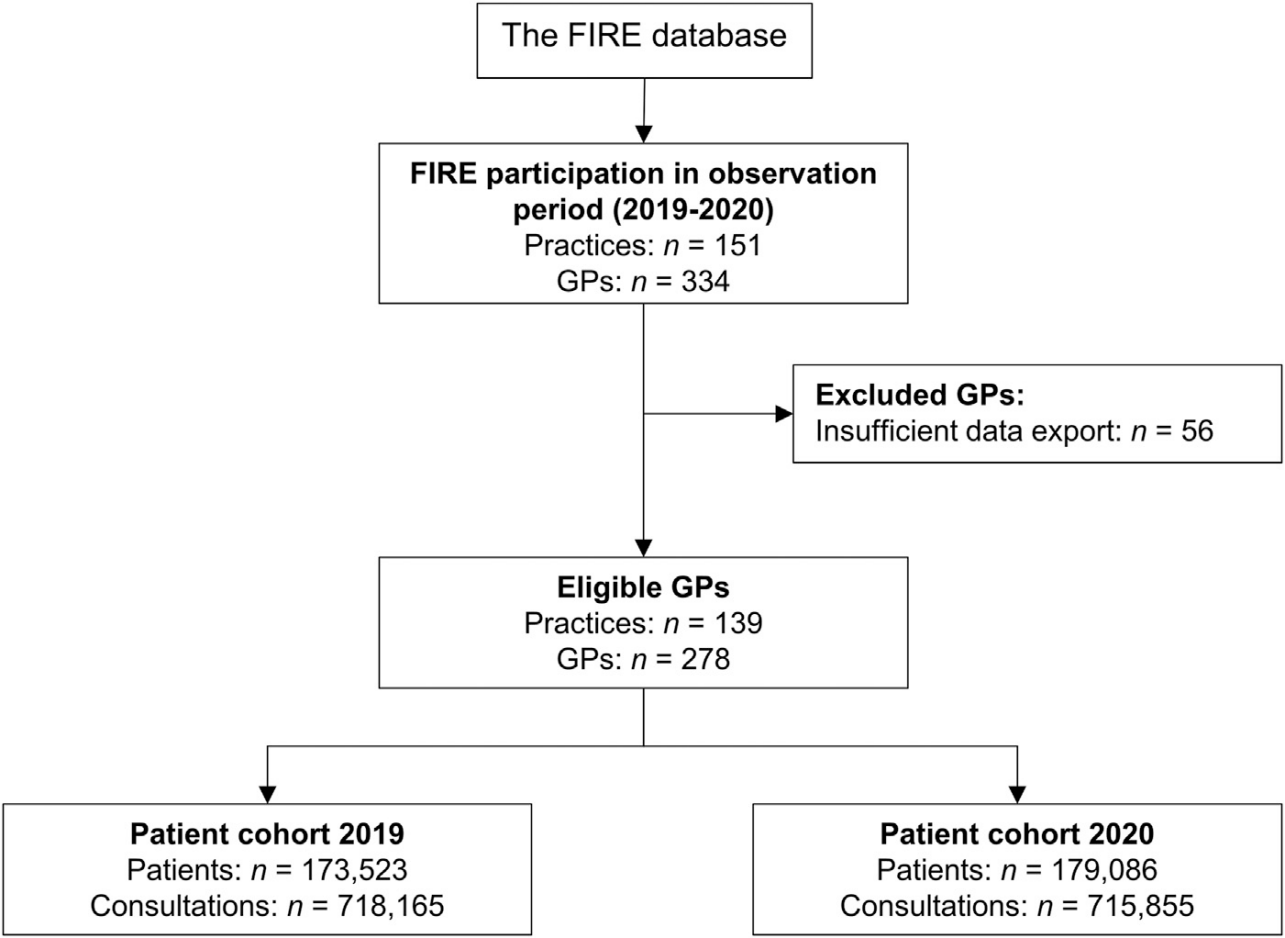
Flowchart of general practitioner and patient selection. Patients of eligible general practitioners were included in the 2019 and/or 2020 cohort if they had at least one consultation before and one consultation during the year of observation. Abbreviations: FIRE, Family medicine International Classification of Primary Care Research using electronic medical records; GP, general practitioner (Impact of the COVID-19 Pandemic on the Intensity of Health Services Use in General Practice: A Retrospective Cohort Study, Switzerland, 2019-2020).

We included GPs who joined the FIRE project prior to the year 2019 and were still participating in 2020. We excluded GPs who did not export data in at least 6 of the first 7 months in both 2019 and 2020. We built two patient cohorts: one in 2019 and one in 2020, with the 2019 serving as reference cohort. Patients were included if they had at least two consultations; one before and one during the year of observation. The study flowchart is shown in Figure 1.

### Database Query and Definitions

We extracted data at practice, GP, and patient levels. For practices, we extracted type of practices (group vs. single). For GPs, we extracted age and sex. For patients, we extracted age, sex, dates of consultations (face-to-face and virtual are indistinguishable in the FIRE database), dates of the first diagnosis (if ever) of diabetes, hypertension, and cardiovascular disease (CVD; for operationalization of these morbidities in the FIRE database, see Supplementary Table 1), and dates and values of measurements of glycated hemoglobin (HbA1c) and blood pressure (BP), as commonly used in diagnosing and monitoring diabetes and hypertension, respectively. Despite several population groups being at high risk for severe COVID-19, in this study, we focused on patients with diabetes, hypertension and CVD, because patients living with these conditions require regular care commonly provided in general practice, and because they are among the most common chronic conditions in Switzerland^5^.

There was no distinct “lockdown period” in Switzerland but rather a stay-at-home recommendation coupled with the closure of non-essential businesses and schools (hereafter referred to as “shutdown period”). For our analyses, we defined this shutdown period as follows: one week after 16 March 2020 (closure of schools and non-essential businesses) to the week before 11 May 2020 (relief of most mitigation measures with a reopening of many non-essential businesses).

### Data Analysis

Data was described by counts (*n*) and proportions (%) or medians and interquartile ranges (IQR). Groups were compared by absolute standardized differences. Missing data above >0.1% was reported. Analyses were performed using the R software version 4.0.0 [15].

Consultation counts were aggregated by calendar week and year, normalized to the total number of patients in the cohort and reported as consultation counts per 100 patients (also referred to as weekly consultation counts). Based on these weekly consultation counts, we built a linear regression model with the year, the season (week as a continuous predictor), holidays (weeks with at least one public holiday) and shutdown period (calendar weeks 13–19) as independent variables. We reported 95% confidence intervals (CI) and *p*-values. The model was then used to predict weekly consultation counts in 2020 that would be expected if there had been no mitigation measures. Fitted values with 95% prediction intervals (PI) were plotted, and observed weekly consultation counts in 2020 were considered significantly different from expected values if they lay outside the PI.

These analyses were repeated for different at-risk subgroups (patients with hypertension, diabetes and CVD) and different age groups (<60 years, 60–80 years, and >80 years), and for measurement counts instead of consultation counts (HbA1c measurements for diabetes, BP measurements for hypertension).

## Results

We observed 173,523 patients in 2019 and 179,086 in 2020; 114,743 patients were part of both cohorts. The total number of different patients amounted to 237,866. A description of patients stratified by year (2019 and 2020) is shown in Table 1. The patients consulted 278 GPs in 139 different practices. Of GPs, 12.6% worked in a single practice and 38.5% were female (0.4% missing). Median GP age was 52 years (IQR = 45–58, 2.5% missing).

**TABLE 1:** Description of patients.

	Cohort 2019 (*n* = 173,523)	Cohort 2020 (*n* = 179,086)	Absolute standardized differences
% Female	54.0	53.6	0.009
Median age at baseline, in years (IQR)	54 (37–70)	54 (37–70)	0.006
% with hypertension	28.4	29.8	0.031
% with diabetes	10.0	11.2	0.036
% with CVD	3.6	4.0	0.023

IQR, interquartile range; CVD, cardiovascular disease. Missing data was below <0.1% (Impact of the COVID-19 Pandemic on the Intensity of Health Services Use in General Practice: A Retrospective Cohort Study, Switzerland, 2019-2020).

### Consultation Counts

The median weekly consultation count per 100 patients over all weeks was 16.9 (IQR = 15.6–17.5) for the total general practice population and 23.2 (IQR = 21.8–24.1) for patients with hypertension, 25.0 (IQR = 22.6–26.3) for patients with diabetes, and 27.6 (IQR = 25.1–28.7) for patients with CVD.

**FIGURE 2:**
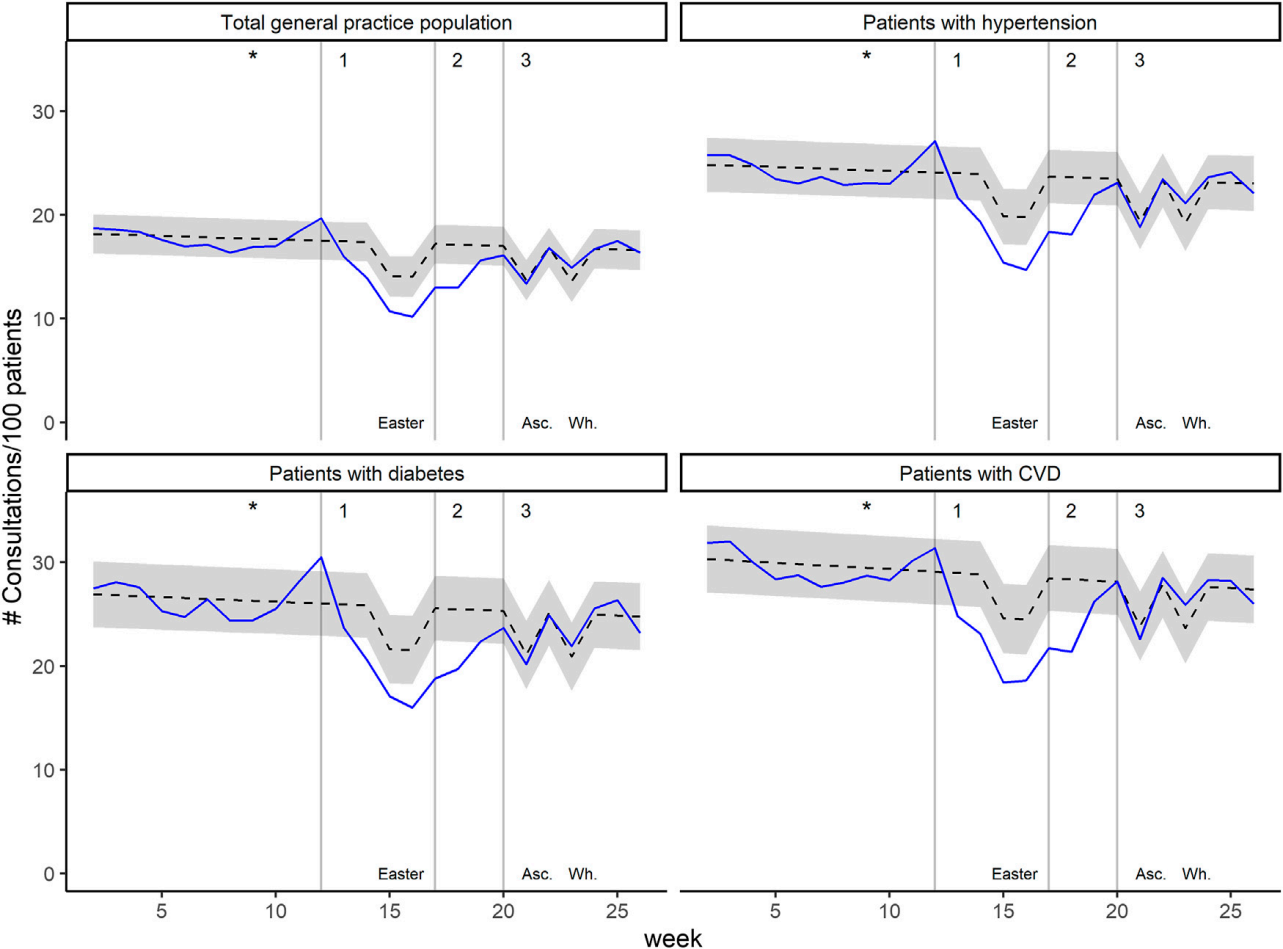
Weekly consultation counts per 100 patients in 2020, for the total general practice population and the at-risk patient groups. Black dashed lines represent predicted values with 95% prediction interval (grey area). Blue lines represent observed values. The asterisks indicate the date of the first confirmed COVID-19 patient in Switzerland. Grey vertical lines indicate 1) introduction of major mitigation measures, 2) relief of ban on non-urgent general practice visits, and 3) relief of most restrictions. Temporary decrease in predicted consultation counts are attributable to public holidays: Easter, Ascension Day (Asc.), and Whitsun (Wh.). (Impact of the COVID-19 Pandemic on the Intensity of Health Services Use in General Practice: A Retrospective Cohort Study, Switzerland, 2019-2020).

**FIGURE 3:**
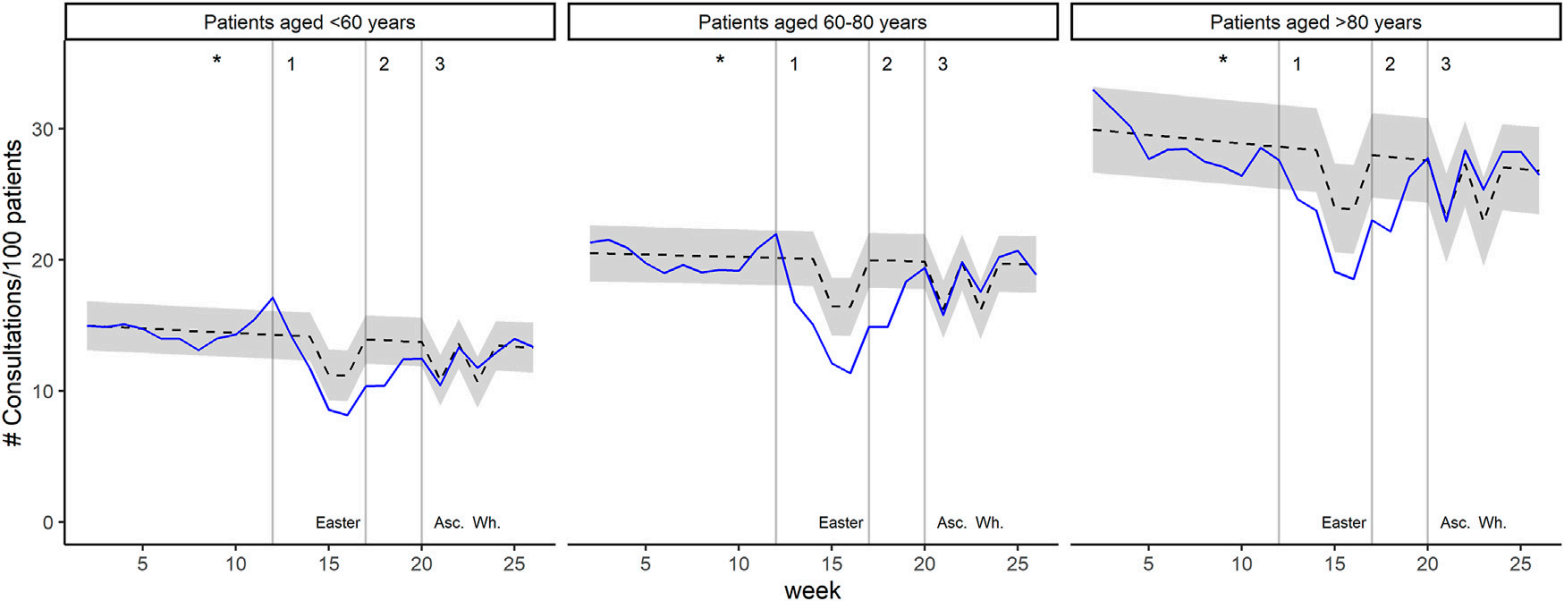
Weekly consultation counts per 100 patients in 2020, for different age groups. Black dashed lines represent predicted values with 95% prediction interval (grey area). Blue lines represent observed values. The asterisks indicate the date of the first confirmed COVID-19 patient in Switzerland. Grey vertical lines indicate 1) introduction of major mitigation measures, 2) relief of ban on non-urgent general practice visits, and 3) relief of most restrictions. Temporary decrease in predicted consultation counts are attributable to public holidays: Easter, Ascension Day (Asc.), and Whitsun (Wh.). (Impact of the COVID-19 Pandemic on the Intensity of Health Services Use in General Practice: A Retrospective Cohort Study, Switzerland, 2019-2020).

During the shutdown period, weekly consultation counts of the total general practice population were 17.2% (*p* < 0.001) lower than expected. The association was similar for patients with hypertension (−16.5%, *p* < 0.001), diabetes (−17.5%, *p* < 0.001) and CVD (−17.6%, *p* < 0.001). For the different age groups, it was −15.7% (*p* < 0.001) for patients aged <60 years, −20.4% (*p* < 0.001) for patient aged 60–80 years, and −14.5% (*p* < 0.001) for patients aged >80 years. Expected and observed weekly consultation counts in 2020 are displayed in Figure 2 (for the total general practice population and the different at-risk patient groups) and Figure 3 (stratification by age groups); see Supplementary Figure 1 for the year 2019. All coefficients with 95% CI and *p*-value are presented in Supplementary Table 2.

### Measurement Counts

The median weekly BP measurement count per 100 patients over all weeks was 2.10 (IQR = 1.76–2.27) for the total general practice population and 3.97 (IQR = 3.46–4.34) for patients with hypertension. For HbA1c, the median weekly measurement count per 100 patients was 0.83 (IQR = 0.68–0.93) for the total general practice population and 3.89 (IQR = 3.27–4.55) for patients with diabetes.

**FIGURE 4:**
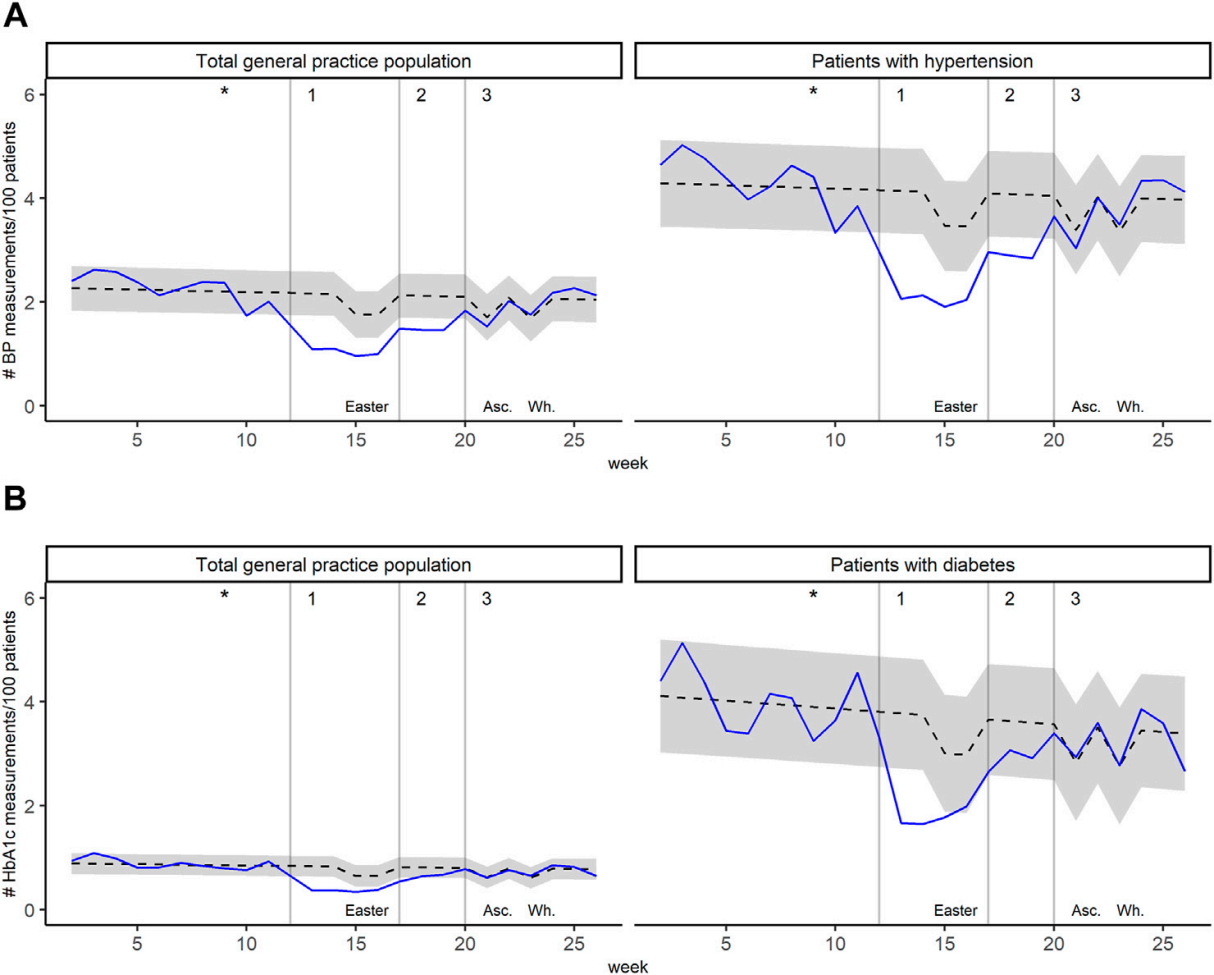
Weekly measurement counts per 100 patients in 2020, for the total general practice population and the relevant condition **(A)** Blood pressure (BP) measurements for the total general practice population and patients with hypertension **(B)** Glycated hemoglobin (HbA1c) measurements for the total general practice population and patients with diabetes. Black dashed lines represent predicted values with 95% prediction interval (grey area). Blue lines represent observed values. The asterisks indicate the date of the first confirmed COVID-19 patient in Switzerland. Grey vertical lines indicate 1) introduction of major mitigation measures, 2) relief of ban on non-urgent general practice visits, and 3) relief of most restrictions. Temporary decrease in predicted consultation counts are attributable to public holidays: Easter, Ascension Day (Asc.), and Whitsun (Wh.). (Impact of the COVID-19 Pandemic on the Intensity of Health Services Use in General Practice: A Retrospective Cohort Study, Switzerland, 2019-2020).

During the shutdown period, weekly BP counts of the total general practice population were −35.3% (*p* < 0.001) lower than expected. The association was similar for patients with hypertension (−35.0%, *p* < 0.001). For HbA1c, weekly counts were −33.2% lower than expected for the total general practice population and −29.8% (*p* < 0001) lower than expected for patients with diabetes. Figure 4 displays expected and observed weekly measurement counts in 2020 for the total general practice population and patients with hypertension (for BP, Figure 4A) or diabetes (for HbA1c, Figure 4B). All coefficients with 95% confidence interval and *p*-value are presented in Supplementary Tables 3, 4.

## Discussion

We found that the period of mitigation measures in Switzerland was associated with a 15%–20% decrease in consultations with GPs and a 30%–35% decrease in HbA1c and BP measurements. Interestingly, proportional decreases were similar for the total general practice population and for patients with chronic conditions at particular high risk for severe COVID-19 (hypertension, diabetes, CVD). Among different age groups, the decrease in consultation counts was highest for patients aged 60–80 and lower for patients aged <60 years and >80 years.

Decreases in health care utilization during the COVID-19 pandemic have been reported in different health care systems and settings. Findings include decreases in consultations to emergency departments [5, 8, 11], psychiatric emergency consultations and number of admissions to psychiatric clinics [7, 10], visits from patients living with HIV [16], non-urgent and surgical ophthalmologic care [9], urologic examinations [6], first diagnoses of diabetes and circulatory system diseases [17], hospitalization for a range of diagnoses [18], as well as different types of preventive and elective care (e.g. mammograms, colonoscopies) [12]. With this study, we could confirm a significant decrease in health care utilization even for Switzerland, which was never in complete lockdown. It is well conceivable that the impact would have been even bigger in case of a complete lockdown.

The decrease in health care utilization may have arisen from a complex interaction between effects related to the shutdown, such as the ban on non-urgent health services, individuals’ fear of contracting the virus, or individuals’ attempts to prevent healthcare services from being overwhelmed [5]. Being more vulnerable to a severe course of disease and bad outcomes, the fear of catching COVID-19 at the GP practice might have been higher for patients in at-risk groups. On the other hand, patients without chronic conditions might have a higher proportion of non-urgent problems, for which medical consultations were banned during the initial phase of the shutdown. Thus, these effects may explain why the proportional reduction in consultations was similar for the general population and patients with hypertension, diabetes, and CVD.

The higher decrease in consultation counts for patients aged 60–80 years might partly be explained by the higher proportion of consultations dedicated to preventive care in this age group compared to the other age groups (<60 years and >80 years)^6^. The consultations dedicated to preventive care were presumably considered non-urgent and postponed to after the shutdown period. In contrast, patients who were most vulnerable (e.g. patients aged >80) were those patients who eventually needed to be seen by their GPs for urgent non-COVID-19-related issues sooner than their younger counterparts. This is in accordance with a British study which found a relative increase in GP consultations with patients with polypharmacy and frailty, concluding that the focus on patients with increased complexity had been retained [19]. Interestingly, we did not observe any signs (in any group) for over-compensation of the “missed” consultations and measurements in the weeks that followed the loosening of the shutdown measures. Instead, our results indicate that after the end of the shutdown, the consultations and measurements picked up in line with the rates that were expected based on the year 2019.

Our second main finding is that the reduction in measurement counts was more pronounced than the reduction in consultation counts. This might be both the result of an increased proportion of virtual consultations [19], which do not allow for any lab and vital sign measurements, as well as a change in reasons of encounter, e.g. shorter consultations focusing on the most prominent problem. Changes in monitoring counts may yield more meaningful insight into changes in regular care than mere changes in consultation counts, especially for patients who require regular monitoring of their chronic conditions (patients with diabetes or hypertension). Therefore, our results suggest that the impact of COVID-19 on regular care (e.g. in patients with chronic disease) might have been even higher than would be expected from the change in consultation counts.

The impact of COVID-19 on regular care poses multiple problems on the health status of patients with chronic conditions, many of whom are at high risk of severe COVID-19. First, the resulting reduction in consultation and measurement rates is in contradiction with comprehensive care based on regular patient–provider interactions. Moreover, staying at home increases the risk for unhealthy diets, decreased physical activity [20, 21], and mental health related concerns [5]. Together, these developments raise concerns of negative medium- and long-term health consequences, which should be the focus of future research.

Future research should also explore patient and GP perspectives around the barriers for receiving continued care from GPs during epidemics. Such research should attempt to disentangle the effects of mitigation measures and fear of contracting coronavirus, particularly for patients at high risk of severe COVID-19 who depend on continued contact with GPs for the treatment of their underlying conditions, and differences in such fears among patient groups.

### Strengths and Limitations

To our knowledge, this is the first study to investigate the impact of COVID-19 on consultation and measurement counts in Swiss general practice. Our careful identification of comparable, large-size populations in the 2019 and 2020 cohorts allowed for a robust investigation of associations between the period of mitigation measures and consultation/measurement counts. The GPs who participate in our database are representative of Swiss ambulatory physicians in terms of age and sex [22]. The additional analysis of patients who are both at high risk of severe COVID-19 and depend on continuous primary health care provided valuable information for these vulnerable patient populations.

This study also has some limitations. First, we were unable to differentiate between consultation types, such as the distinction between virtual and face-to-face consultations, which most likely shifted during the pandemic [19]. We assumed, however, that every type of encounter was entered in the electronic health records, allowing us to reliably capture the actual health services use. Moreover, BP and HbA1c measurements were presumably performed in the practice and are thus indicative of face-to-face consultations. Second, we were not able to differentiate whether consultations were prompted by the GP or the patient, and we had no information on patients’ reasons for consulting with GPs. Future research should investigate this issue further. Knowing reasons for encounter would also enable a differentiation between routine consultations and COVID-19-related consultations. However, drawing on measurement counts gave some insight into this matter. Third, we faced limitations that are inherent to database studies, such as missing data entries for diagnosis codes and laboratory and vital signs measurements. We do not, however, expect missing rates to change over time. Therefore, our time trend analysis should not be majorly affected by this limitation.

### Conclusion

We found relevant decreases in consultation counts and even larger decreases in chronic disease monitoring during the period of mitigation measures, for the total general practice population, for different age groups, and for patients with selected chronic conditions who are considered at high risk of severe COVID-19-related complications. Comprehensive, regular care is crucial for patients with chronic conditions. Although the priority is undoubtedly to contain the spread and impact of COVID-19 while securing health care for a potentially larger proportion of COVID-19 patients, it remains nonetheless vital that health care systems are able to continue meeting the needs of the entire population, and particularly vulnerable populations, during pandemic settings. This requires a careful balance between protecting GPs and patients against COVID-19 infections and preventing the rapid deterioration in patients with chronic conditions.

## Data Availability

The data analyzed in this study is subject to the following licenses/restrictions: The data was gathered within the ongoing FIRE project. The FIRE database can be accessed at any time by the scientific team of the institute. Requests to access these datasets should be directed to Thomas Rosemann, thomas.rosemann@usz.ch.

## Footnotes

1 Federal Office of Public Health. New coronavirus: Measures, ordinance and explanations (2020) [08.05.2020]. Available from: https://www.bag.admin.ch/bag/de/home/krankheiten/ausbrueche-epidemien-pandemien/aktuelle-ausbrueche-epidemien/novel-cov/massnahmen-des-bundes.html.

2 Swiss medical association FMH. [Non-urgent treatments according to COVID-19 regulation 2] (2020) [09.04.2020]. Available from: https://www.fmh.ch/dienstleistungen/kommunikation/aktuelle-infos-coronavirus.cfm#i139521.

3 Federal Office of Public Health. [New corona virus: At risk groups] (2020). Available from: https://www.bag.admin.ch/bag/de/home/krankheiten/ausbrueche-epidemien-pandemien/aktuelle-ausbrueche-epidemien/novel-cov/besonders-gefaehrdete-menschen.html.

4 Braunmiller H./Sanitas [COVID-19: No fear of family doctor and hospital] (2020). Available from: https://www.sanitas.com/de/magazin/zusammenleben-heute/trotz-coronakrise-zum-hausarzt.html.

5 Federal Office of Public Health. [Numbers and facts on non-communicable diseases] (2020) [13.11.2020]. Available from: https://www.bag.admin.ch/bag/de/home/zahlen-und-statistiken/zahlen-fakten-nichtuebertragbare-krankheiten.html.

6 EviPrev. [Evidence-based prevention in primary care: Products & tools. EpiPrev recommendations] (2019) [13.11.2020]. Available from: https://eviprev.ch/produkte-tools/.
