# Supplementary material for: Impact of the COVID-19 Pandemic on the Intensity of Health Services Use in General Practice: A Retrospective Cohort Study
Source: Int J Public Health. 2021 May 7;66:635508. doi: 10.3389/ijph.2021.635508 (PMC8565270; doi:10.3389/ijph.2021.635508)
Supplement: Supplementary file 1 [file DataSheet1.pdf]

Supplementary Material

Supplementary Table 1. Operationalization of morbidities

Chronic condition	Operationalization
Cardiovascular disease	Any of the following before the observation period:<br><br>- ICPC-2 = K74 | K75 | K76 | K89 | K90 | K91 | K92<br>- ATC code B01AC04 | B01AC22 | B01AC25 | B01AC24
Diabetes	Any of the following before the observation period:<br><br>- ICPC-2 = T89 | T90<br>- Glycated hemoglobin > 6.5%<br>- ATC code ^A10
Hypertension	Any of the following before the observation period:<br><br>- ICPC-2 = K85 | K86 | K87<br>- two blood pressure measurements > 140/90 mmHg<br>- ATC code ^C02 | ^C03A | ^C03EA01 | ^C07 | ^C08 | ^C09A | ^C09B

ICPC-2, international classification of primary care 2nd version; |, logical "OR"; ^, starting with ….; ATC, anatomical technical chemical

Supplementary Table 2. Regression results for weekly consultation counts/100 patients

a) Total general practice population and patients with selected chronic conditions

Variable	Total general practice population			Patients with Hypertension			Patients with Diabetes			Patients with CVD		
	Est (%)	95% CI	*p*-value	Est (%)	95% CI	*p*-value	Est (%)	95% CI	*p*-value	Est (%)	95% CI	*p*-value
Intercept	18.3 (100)	17.7 to 18.9	<0.001	25 (100)	24.1 to 25.9	<0.001	27.1 (100)	26 to 28.2	<0.001	30.6 (100)	29.5 to 31.6	<0.001
Shutdown	-3.1 (-17.2)	-4 to -2.3	<0.001	-4.1 (-16.5)	-5.3 to -3	<0.001	-4.7 (-17.5)	-6.1 to -3.4	<0.001	-5.4 (-17.6)	-6.8 to -4	<0.001
Holidays	-3.2 (-17.6)	-4 to -2.5	<0.001	-4 (-16)	-5 to -3	<0.001	-4.1 (-15.2)	-5.4 to -2.9	<0.001	-4.1 (-13.5)	-5.4 to -2.9	<0.001
Week (Season)	-0.1 (-0.4)	-0.1 to 0	0.001	-0.1 (-0.3)	-0.1 to 0	0.005	-0.1 (-0.3)	-0.2 to 0	0.006	-0.1 (-0.4)	-0.2 to -0.1	<0.001
Year (2019 compared to 2020)	-0.3 (-1.7)	-0.9 to 0.2	0.262	-0.3 (-1.2)	-1.1 to 0.5	0.423	-0.4 (-1.4)	-1.3 to 0.6	0.410	-1.1 (-3.6)	-2.1 to -0.1	0.025

CVD; cardiovascular disease; Est (%), estimates (percentages with respect to the intercept); CI, confidence interval

Supplementary Table 2. Regression results for weekly consultation counts/100 patients

b) Patients in different age groups

Variable	< 60 year old patients			60-80 year old patients			>80 year old patients		
	Est (%)	95% CI	*p*-value	Est (%)	95% CI	*p*-value	Est (%)	95% CI	*p*-value
Intercept	15.1 (100)	14.5 to 15.7	<0.001	20.6 (100)	19.9 to 21.3	<0.001	30.2 (100)	29.1 to 31.3	<0.001
Shutdown	-2.4 (-15.7)	-3.2 to -1.6	<0.001	-4.2 (-20.4)	-5.1 to -3.3	<0.001	-4.4 (-14.5)	-5.8 to -3	<0.001
Holidays	-2.8 (-18.9)	-3.6 to -2.1	<0.001	-3.6 (-17.5)	-4.4 to -2.8	<0.001	-4.3 (-14.1)	-5.5 to -3	<0.001
Week (Season)	-0.1 (-0.5)	-0.1 to 0	<0.001	0 (-0.2)	-0.1 to 0	0.096	-0.1 (-0.4)	-0.2 to -0.1	<0.001
Year (2019 compared to 2020)	-0.3 (-2.1)	-0.9 to 0.2	0.254	-0.3 (-1.4)	-0.9 to 0.3	0.345	-0.3 (-1)	-1.3 to 0.7	0.532

Est (%), estimates (percentages with respect to the intercept); CI, confidence interval

Supplementary Table 3. Regression results for weekly blood pressure measurement counts/100 patients

Variable	All patients			Patients with hypertension		
	Est (%)	95% CI	*p*-value	Est (%)	95% CI	*p*-value
Intercept	2.28 (100)	2.14 to 2.43	<0.001	4.31 (100)	4.03 to 4.6	<0.001
Shutdown	-0.81 (-35.3)	-0.99 to -0.62	<0.001	-1.51 (-35.0)	-1.87 to -1.15	<0.001
Holidays	-0.38 (-16.7)	-0.55 to -0.21	<0.001	-0.64 (-14.9)	-0.97 to -0.32	<0.001
Week (Season)	-0.01 (-0.4)	-0.02 to 0	0.036	-0.01 (-0.3)	-0.03 to 0	0.113
Year (2019 compared to 2020)	0 (-0.1)	-0.13 to 0.13	0.973	-0.04 (-1)	-0.29 to 0.2	0.723

Est (%), estimates (percentages with respect to the intercept); CI, confidence interval

Supplementary Table 4. Regression results for weekly glycated haemoglobin measurement counts/100 patients

	All patients			Patients with diabetes		
	Est (%)	95% CI	*p*-value	Est (%)	95% CI	*p*-value
Intercept	0.9 (100)	0.83 to 0.96	<0.001	4.17 (100)	3.81 to 4.54	<0.001
«Lockdown»	-0.3 (-33.2)	-0.38 to -0.21	<0.001	-1.24 (-29.8)	-1.71 to -0.78	<0.001
Holidays	-0.18 (-19.7)	-0.25 to -0.1	<0.001	-0.71 (-17)	-1.13 to -0.29	0.002
Week (Season)	0 (-0.5)	-0.01 to 0	0.025	-0.03 (-0.7)	-0.05 to -0.01	0.007
Year (2019 compared to 2020)	0.08 (8.6)	0.02 to 0.14	0.012	0.69 (16.6)	0.37 to 1.01	<0.001

Est (%), estimates (percentages with respect to the intercept); CI, confidence interval

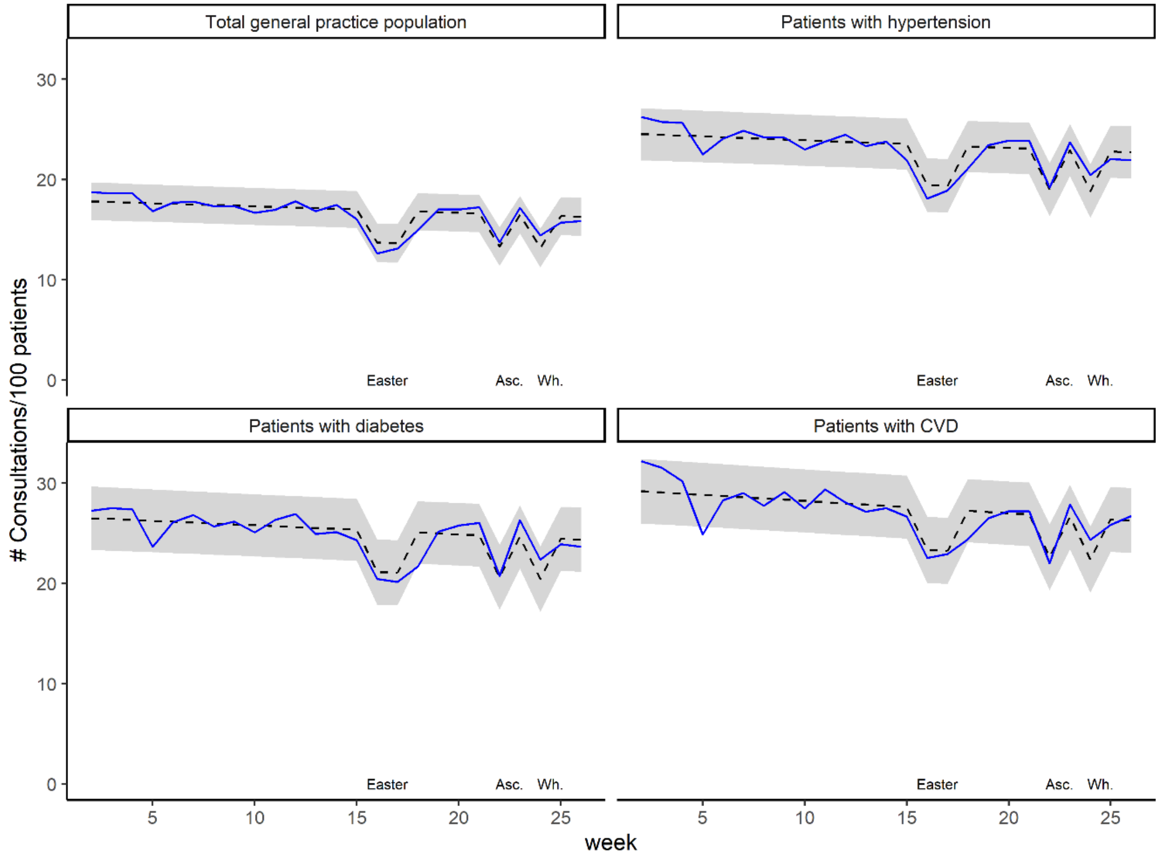

Supplementary Figure 1. Weekly consultation counts per 100 patients in 2019, for the total general practice population and the at-risk patient groups. Black dashed lines represent predicted values with 95% prediction interval (grey area), blue lines represent observed values. Temporary decrease in predicted consultation counts are attributable to public holidays: Easter, Ascension Day (Asc.), and Whitsun (Wh.)
